# Quality of life following hip fractures: results from the Norwegian hip fracture register

**DOI:** 10.1186/s12891-016-1111-y

**Published:** 2016-07-07

**Authors:** Jan-Erik Gjertsen, Valborg Baste, Jonas M. Fevang, Ove Furnes, Lars Birger Engesæter

**Affiliations:** Department of Orthopaedic Surgery, Haukeland University Hospital, Jonas Lies vei 65, N 5021 Bergen, Norway; Department of Clinical Medicine, Faculty of Medicine and Odontology, University of Bergen, Bergen, Norway

**Keywords:** Health related quality of life, Hip fractures, Orthopaedic surgery, Femoral neck fractures, National results

## Abstract

**Background:**

Patient-reported health-related quality of life is an important outcome measure when assessing the quality of hip fracture surgery. The frequently used EQ-5D index score has unfortunately important limitations. One alternative can be to assess the distribution of each of the five dimensions of the patients’ descriptive health profile. The objective of this paper was to investigate health-related quality of life (HRQoL) after hip fractures.

**Methods:**

Data from hip fracture operations from 2005 through 2012 were obtained from The Norwegian Hip Fracture Register. Patient reported HRQoL, (EQ-5D-3L) was collected from patients preoperatively and at four and twelve months postoperatively *n* = 10325. At each follow-up the distribution of the EQ-5D-3L and mean pain VAS was calculated.

**Results:**

Generally, a higher proportion of patients reported problems in all 5 dimensions of the EQ-5D-3L at all follow-ups compared to preoperative. Also a high proportion of patients with no preoperative problems reported problems after surgery; At 4 and 12 months follow-ups 71 % and 58 % of the patients reported walking problems, and 65 % and 59 % of the patients reported pain respectively. Patients with femoral neck fractures and the youngest patients (age < 70 years) reported least problems both preoperatively and at all follow-ups.

**Conclusions:**

A hip fracture has a dramatic impact on the patients’ HRQoL, and the deterioration in HRQoL sustained also one year after the fracture. Separate use of the descriptive profile of the EQ-5D is informative when assessing quality of life after hip fracture surgery.

**Electronic supplementary material:**

The online version of this article (doi:10.1186/s12891-016-1111-y) contains supplementary material, which is available to authorized users.

## Background

Osteosynthesis of hip fractures, and in particular the displaced fractures of the femoral neck (FFN), has been associated with a high risk of reoperations [1–5]. In the later years, however, there has been a trend towards primary arthroplasty for the displaced FFNs [6, 7] and accordingly the number of reoperations for these specific fractures has decreased [6]. Nevertheless, for the individual patients any major reoperation represents a temporary increase in both morbidity and mortality. The number of reoperations has traditionally been the most common way of reporting the outcome after hip fracture surgery. The recent decades, however, an increasing number of studies on hip fractures have focused also on other outcome variables, such as functional outcome and patient-reported outcome measures (PROM) [1, 3, 8–10]. The importance of such PROM data when measuring the quality of surgery in orthopaedic studies has been advocated by several authors [11, 12].

It is well-known that a hip fracture has impact on patients’ quality of life [1, 3, 8, 9, 13–17]. Most studies that have used the EQ-5D-3L as an instrument for measuring quality of life have used the EQ-5D index score, which is a weighted value that can be calculated from different tariffs with adjustments for cultural and national differences. Several studies have lately reported important limitations of this index score, such as bimodal or trimodal distribution and a ceiling effect [18–20]. One other disadvantage of the EQ-5D index-score is that this single value does not provide information on in which way the patients’ quality of life is reduced. To get as much information as possible from the EQ-5D data one alternative can be to investigate and report separately the distribution of each of the five dimensions of health-related quality of life; mobility, self-care, usual activities, pain/discomfort, and anxiety/depression, as presented in this study.

The Norwegian Hip Fracture Register (NHFR) has recorded hip fractures on a national level since 2005 [21]. Besides data on reoperations and mortality, the NHFR also provides PROM data including the EQ-5D-3L questionnaire. Based on data from the NHFR we aimed to investigate the changes in quality of life associated with hip fractures.

## Methods

The NHFR collects data on hip fractures in Norway as a prospective observational study. Compared with the Norwegian Patient Registry, the completeness of the registration has earlier been found to be approximately 89 % [6]. The Norwegian Data Inspectorate approved the recording of data. All patients signed an informed consent form that was entered into their hospital medical record. Data on each primary operation for hip fractures are reported on standard one-page forms to the register by the surgeon. The form includes information on the patients (age, sex, cognitive function, and ASA-class [22]), the fracture, and the operation. A more thoroughly description of the NHFR has been published earlier [21]. In the present study the fractures were categorized into three groups: intracapsular fractures of femoral neck (FFN), trochanteric fractures (including basocervical fractures), and subtrochanteric fractures (including AO/AAOS A3 “Intertrochanteric” fractures).

The patients received questionnaires directly from the register 4 and 12 months postoperatively. These questionnaires included the Norwegian translation of the Euroqol [23]. The Euroqol is a standardized non-disease-specific tool for describing the health–related quality of life. Both the health status part (EQ-5D-3L) and the visual analogue scale (EQ-VAS) were filled in by the patients. The EQ-5D-3L is based on five dimensions of health-related quality of life; mobility, self-care, usual activities, pain/discomfort, and anxiety/depression. Each item has three levels of severity; no problems, some problems, or major problems. The EQ-5D data in this article are presented as health profiles from this descriptive system. The preoperative EQ-5D health profile was reported as part of the four-month questionnaire, and consequently retrospectively recorded by the patients. Furthermore, the questionnaires included a visual analogue scale (VAS 0-100) where the patients reported the average level of pain from the operated hip during the last months (with 0 indicating no pain and 100 indicating extreme pain).

### Study sample

**Fig. 1:**
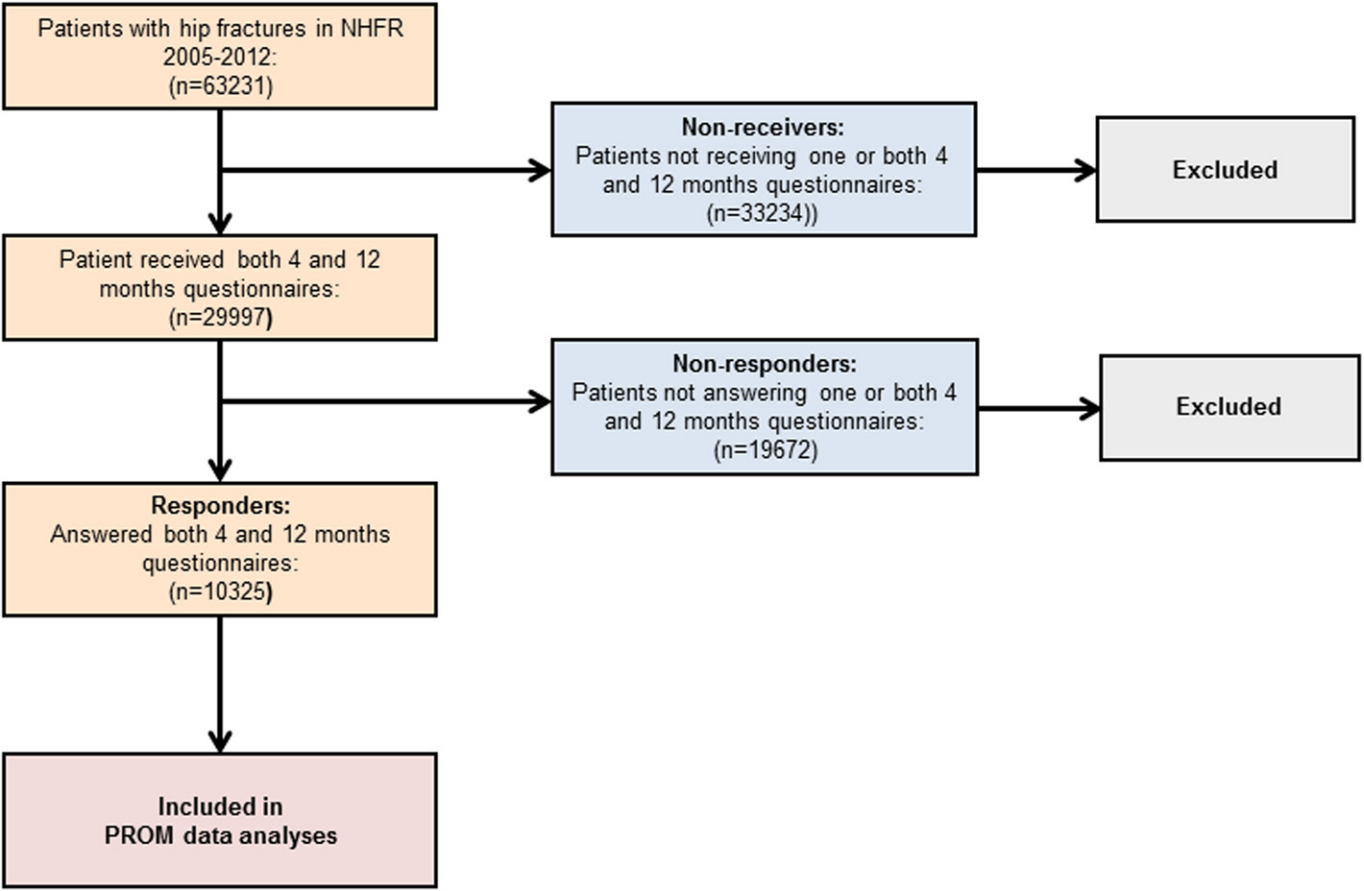
Flow chart of patients included in the study

Patients operated due to an acute hip fracture and reported to the NHFR from 2005 to 2012 were eligible for inclusion in the present study. As of December 31, 2012 there were 63,231 hip fractures recorded in the NHFR. The four months questionnaire had been sent to 37,968 patients and the twelve months questionnaire had been sent to 30,400 patients. The response rates to the questionnaires were 54 % at four months and 49 % at twelve months. Only patients who had received and completely filled in both the four- and twelve months questionnaires were included in the study. Patients who died before time of the planned follow-up and patients with too short follow-up did not received questionnaires. Further, due to economical/administrative reasons only a randomly selected group of patients in the time period 2007-2009 received the questionnaires. A total of 29,997 patients received both questionnaires. Of these patients 10,324 (34 %) answered both questionnaires completely, and were accordingly included in the study (Fig. 1).

The baseline characteristics for responders and non-responders are presented in Table 1. The responders were statistically significant younger, healthier according to the ASA classification, and less cognitively impaired compared to the non-responders. Further, there were small, but still statistically significant differences both in type of fracture and type of surgery (Table 1).

**Table 1:** Baseline characteristics for responders and non-responders^a^

	Responders (*n* = 10325)	Non-responders (*n* = 19672)	*P* value
Mean age (yrs) (SD)	77.3 (11.7)	79.8 (11.7)	<0.001*
Female (%)	7471 (72.4)	14420 (73.3)	0.097**
ASA^b^-class (%)			<0.001**
1	1402 (13.6)	1420 (7.2)	
2	4525 (43.8)	7262 (36.9)	
3	3964 (38.4)	9878 (50.2)	
4	261 (2.5)	791 (4.0)	
5	1 (0)	11(0.1)	
Data missing	172 (1.7)	310 (1.6)	
Cognitive impairment (%)			<0.001**
No	8267 (80.1)	12581 (64.9)	
Yes	834 (8.1)	4290 (22.1)	
Uncertain	659 (6.4)	2090 (10.8)	
Data missing	565 (5.5)	431 (2.2)	
Fracture type (%)			<0.001**
Femoral neck fracture	5639 (54.6)	10236(52.0)	
Trochanteric fracture	3706 (35.9)	7635 (38.8)	
Subtrochanteric fracture	875 (8.5)	1586 (8.1)	
Other/missing	105 (1.0)	215 (1.1)	
Primary operation (%)			<0.001**
Screws/pins	2643 (25.6)	4638 (23.6)	
Hemiarthroplasty	2567 (24.9)	5161 (26.2)	
Total hip arthroplasty	382 (3.7)	280 (1.4)	
Sliding hip screw	3154 (30.5)	6466 (32.9)	
Intramedullary nail	1249 (12.1)	2539 (12.9)	
Other	330 (3.2)	588 (3.0)	

^a^Responders: patients who completely answered both the 4 and 12 months questionnaires; Non reseponders: patients who received both the 4 and 12 months questionnaire but did not completely answered one or both questionnaires

^b^ASA, American Society of Anaesthesiologists

*independent *t*-test

**Pearson chi-squared test

### Statistical analysis

The results are presented as number and/or percent of patients reporting quality of life in each level of the five EQ-5D dimensions. The Pearson chi-squared test was used for comparison of categorical variables and the independent t-test was used for continuous variables. We performed sub-analyses for each of the five dimensions including only patients reporting no problems preoperatively. Further, separate analyses were done for different fracture types (FFN, trochanteric fracture, and subtrochanteric fracture) and for different age groups (<70 years, 70–80 years, and > 80 years). We did not adjust for patients who were operated on both sides. The significance level was set to 0.05 and all *p* values were two-tailed. The statistical analyses were performed in the statistical package IBM SPSS statistics version 21 (SPSS Inc., Chicago, IL).

## Results

### Quality of life

Preoperatively, the majority of the patients reported no problems in each of the five dimensions of the EQ-5D (Table 2). Compared to their preoperative status, the proportion of patients reporting problems at four months more than doubled in the dimensions regarding mobility and self-care, and almost doubled regarding usual activities and pain/discomfort. At twelve months postoperatively there was still a marked increase of patients reporting problems in these dimensions compared to preoperatively. For the last dimension (Anxiety/depression) the changes were less evident.

**Table 2:** Descriptive profile of the 5 dimensions of EQ-5D after hip fracture. All patients included

	Preoperative	4 months postop	12 months postop
	*n* (%)	*n* (%)	*n* (%)
Mobility			
No problems in walking about	6462 (62.6)	2039 (19.7)	3203 (31.0)
Some problems in walking about	3750 (36.3)	7991 (77.4)	6795 (65.8)
Confined to bed	113 (1.1)	295 (2.9)	327 (3.2)
Self-care			
No problems with self-care	8013 (77.6)	5434 (52.6)	6120 (59.3)
Some problems with self-care	1866 (18.1)	2882 (37.6	3246 (31.4)
Unable to wash or dress	446 (4.1)	1009 (9.8)	959 (9.3)
Usual activities			
No problems in performing usual activities	6217 (60.2)	2619 (25.4)	3418 (33.1)
Some problems in performing usual activities	3098 (30.0)	5604 (54.3)	4880 (47.3)
Unable to perform usual activities	1010 (9.8)	2102 (20.4)	2027 (19.6)
Pain/discomfort			
No pain or discomfort	6446 (62.4)	2612 (25.3)	3534 (34.2)
Some pain or discomfort	3354 (32.5)	6779 (65.7)	6065 (58.7)
Extreme pain or discomfot	525 (5.1)	934 (9.0)	726 (7.0)
Anxiety/depression			
Not anxious or depressed	7636 (74.0)	6476 (62.7)	6549 (63.4)
Moderately anxious or depressed	2412 (23.4)	3406 (33.0)	3411 (33.0)
Exteremely anxious or depressed	277 (2.7)	443 (4.3)	365 (3.5)

When performing sub-analyses for each of the five EQ-5D dimensions including only patients with no reported problems preoperatively, there was still a high proportion of patients reporting problems after four and twelve months (Table 3). In the group of patients reporting no problems in walking preoperatively 71 % reported problems after 4 months and 58 % had problems after 12 months postoperatively. Corresponding results were found for the ability of performing self-care and for usual activities where 29 % and 53 % respectively reported problems twelve months postoperatively. As much as 60 % of the patients with no preoperative pain reported pain twelve months postoperative.

**Table 3:** Descriptive profile of the 5 dimensions of EQ-5D after hip fracture. Sub-analyses including only patients reporting no problems preoperatively

	4 months	12 months
	postop	postop
	*n* (%)	*n* (%)
Mobility (*n* = 6462)		
No problems in walking about	1858 (28.8)	2699 (41.8)
Some problems in walking about	4544 (70.3)	3702 (57.3)
Confined to bed	60 (0.9)	61 (0.9)
Self-care (*n* = 8013)		
No problems with self-care	5197 (64.9)	5696 (71.1)
Some problems with self-care	2601 (32.5)	2097 (26.2)
Unable to wash or dress	215 (2.7)	220 (2.7)
Usual activities (*n* = 6217)		
No problems in performing usual activities	2430 (39.1)	2953 (47.5)
Some problems in performing usual activities	3309 (53.2)	2831 (45.5)
Unable to perform usual activities	478 (7.7)	433 (7.0)
Pain/discomfort (*n* = 6446)		
No pain or discomfort	2252 (34.9)	2650 (41.1)
Some pain or discomfort	3845 (59.6)	3507 (54.4)
Extreme pain or discomfot	349 (5.4)	289 (4.5)
Anxiety/depression (*n* = 7636)		
Not anxious or depressed	6197 (81.2)	5852 (76.6)
Moderately anxious or depressed	1366 (17.9)	1691 (22.1)
Exteremely anxious or depressed	73 (1.0)	93 (1.2)

For each dimension, only patients who reported «no problem» preoperatively are included

### PROM data according to fracture type

**Fig. 2:**
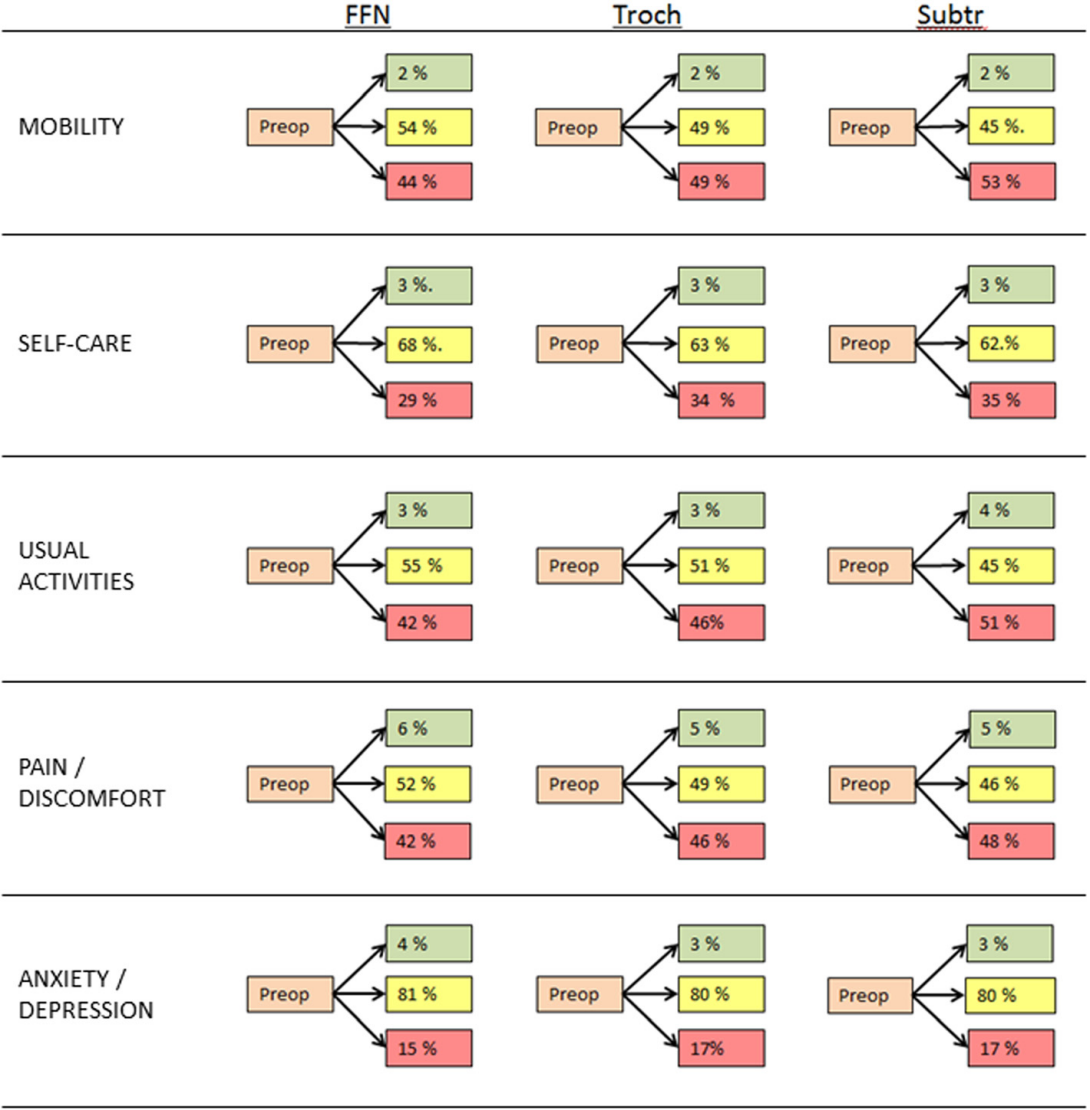
Changes in EQ5D from preoperative to 4 months postoperatively. Changes in severity level in each of the five dimensions of the EQ-5D for different fracture types. Green indicates improvement/less problems, yellow indicates no change, and red indicates worsening/more problems

The quality of life by the EQ-5D proportions for the different fracture types is presented in Additional file 1. The patients operated due to trochanteric fractures reported statistically significant more problems preoperatively than the other fracture types. The patients operated due to a FFN reported statistically significant lesser problems at all follow-ups compared to other fracture types. Regarding anxiety and depression the differences were less evident, but still better results were reported for the FFNs. The changes in severity level from preoperative to the four months follow up for each of the dimensions of the EQ-5D and for each fracture type are shown in Fig. 2. More than half of the patients with FFN reported no changes in the severity level of each dimension at four months postoperatively compared to their preoperative quality of life. Compared to patients with FFN, a higher proportion of patients with trochanteric and in particular subtrochanteric fractures reported increased problems in all dimensions at four months postoperative. For all fracture types only a small proportion of patients reported less problems four months postoperative compared to their preoperative functional level.

**Fig. 3:**
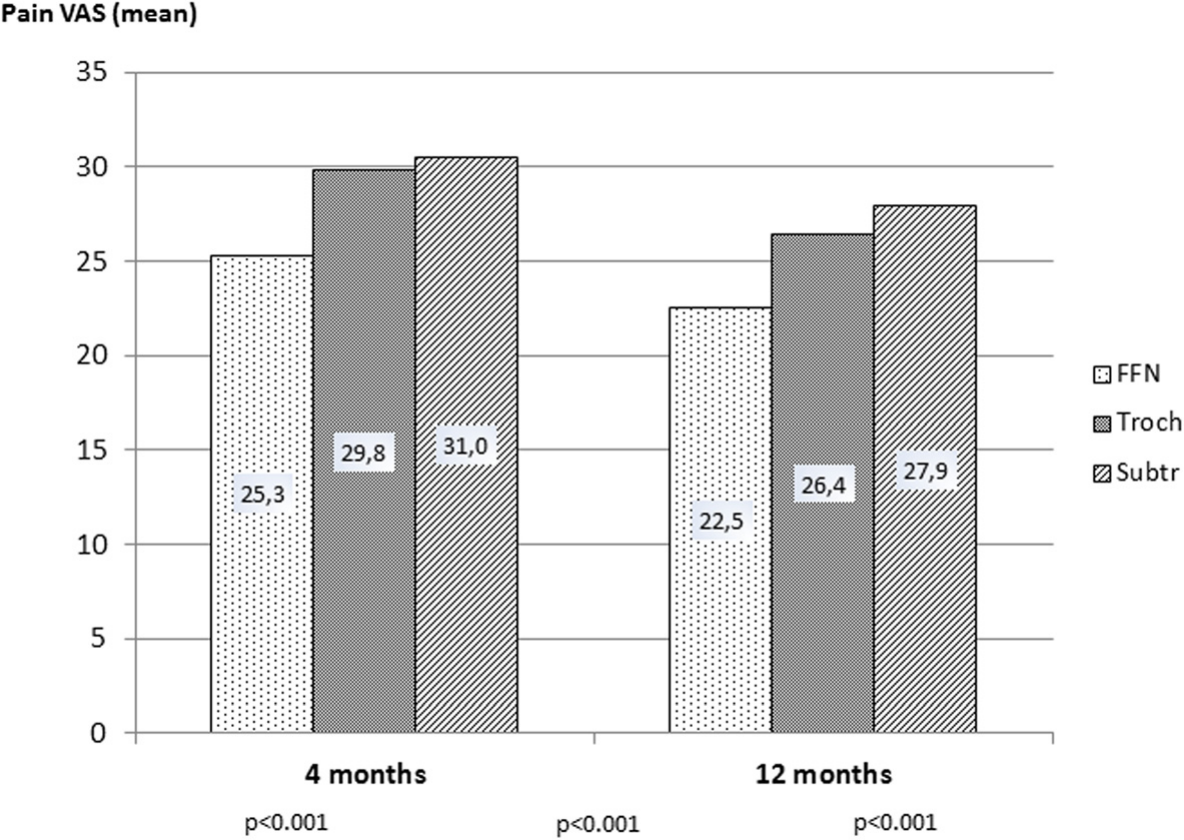
Mean pain from the operated hip at different follow-ups according to fracture type. Visual analogue scale, (VAS) 0-100 where 0 indicating no pain and 100 indicating unbearable pain. P-values were assessed using the ANOVA

Figure 3 shows the mean VAS pain from the operated hip at the two postoperative follow-ups. Differences in mean pain between the different fracture types were found at all follow-ups; i.e. patients operated for a FFN reported the lowest pain at all follow-ups compared to trochanteric and subtrochanteric fractures. For all fracture types the mean pain decreased over time. However, the mean pain after 12 months was still between 22 and 28, indicating that pain from the operated hip still may be an issue for at least some of the patients.

### PROM data according to age

The youngest age group (<70 years) reported the best quality of life in all dimensions except pain/discomfort at all follow-ups compared to the older age groups [see Additional file 2]. However, even in the youngest patient group problems in all dimensions were frequent following hip fractures. After twelve months more than 56 % of the youngest patients had problems in walking, 23 % had problems with self-care, and 51 % had problems performing usual activities. The oldest age group (>80 years) reported problems most frequently. The differences between the age groups were statistically significant in all dimensions and at all follow-ups.

## Discussion

To our knowledge this is the first study presenting complete descriptive EQ-5D health profiles for a large group of patients with hip fractures on a national level. The quality of life according to the EQ-5D was considerable reduced after a hip fracture and the deterioration sustained the first year postoperatively. The changes in EQ-5D were present in all age groups and for all types of fracture. The most interesting finding was, however, that also a lot of patients reporting no preoperative problems in walking, with self-care, and in performing usual activities experienced the same deterioration in function.

The deterioration in quality of life after hip fractures found in the present article is in good accordance with earlier results from both randomized trials and prospective studies [1–3, 5, 24–26]. Most studies reporting quality of life results have used the EQ-5D index score. As these scores can be based on different tariffs, with adjustments for cultural and national differences, the values presented in different studies may not be directly comparable.

The patients with femoral neck fractures reported less problems, higher quality of life, and lower average pain from the operated hip at all follow-ups compared to those with trochanteric or with subtrochanteric fractures. In Norway, there has been a change from closed reduction and internal fixation towards extensive use of hemiarthroplasties in the treatment of displaced femoral neck fractures [6]. Hemiarthroplasties have in earlier studies been found to result in fewer reoperations and provide better functional results than internal fixation for femoral neck fractures [1, 2, 27]. Accordingly, treatment with hemiarthroplasty is probably one reason to the good PROM outcomes for the femoral neck fractures in the present study. Patients with trochanteric fractures reported more problems and pain preoperatively compared to the other fracture types. The reason for this is unclear. However, one contributing factor can be that patients with trochanteric fractures earlier have been found to be older than patients with other fracture types [21, 28].

One interesting finding in this study was that a surprisingly high proportion of the patients reported no changes in the severity levels of EQ-5D-3L at four months postoperatively compared to their preoperative levels. These results probably illustrate one important limitation of the EQ-5D-3L. With only three severity levels the discriminatory power may be too low. When analyzing the results of hip fracture patients, one should have in mind that a high proportion of these patients have a reduced walking ability, have problems with self-care and in performing usual activities, and are suffering from pain or discomfort already before the hip fracture. Consequently, the EQ-5D-3L instrument may have problems in detecting further deterioration in quality of life.

### Strength and limitations

The strengths of our results are the high number of patients and that we present nation-wide results. The response rates of the 4 and the 12 months questionnaires were approximately 50 % for the living patients, and the response rate of patients answering both questionnaires was only 34 %. The reason for this low response rate is probably high age and high degree of comorbidity among the patients. Reminders could probably have improved the response rate. The study population represented a selected group of patients as they have all survived the first 12 months after surgery and answered the 12 months questionnaire. The results showed that they were younger and healthier than the non-responders. This is also verified when comparing the baseline characteristics of patients in the present study with earlier studies from the Norwegian Hip Fracture Register, which has reported on older and more comorbid patients [2, 29–33]. Accordingly, one major limitation of the present study is the risk for selection bias. Thus, the results reported in this study may be a best-case scenario excluding the oldest and most comorbid patients with the expected worst quality of life. However, even if a selection bias exists, the absolute number of patients reporting problems following hip fractures was still high.

One other limitation of the study is that it was not a randomized trial and, accordingly, no matched control group. We cannot conclude that all the changes in quality of life over time were caused by the hip fracture itself. To some extent these changes were probably part of the natural life course for these old and frail patients irrespective of the fracture. The EQ-5D index score has been thoroughly validated in several studies including elderly hip fracture patients [34–38]. As far as we know, similar validation of the EQ-5D health profiles has not been done. However, the use of EQ-5D health profiles as used in the present study is one of the recommended methods to present quality of life results according to the EuroQol group [39]. Finally, a recall bias may exist as the preoperative EQ-5D data was retrospectively recorded four months after surgery. Two studies have found moderate or good correlation when comparing recalled data and prospective data in outcome studies after arthroplasties [40, 41]. Consequently we believe we largely can trust the recalled preoperative data.

## Conclusions

A hip fracture has a dramatic impact on the patients’ HRQoL, also for patients with no health-related problems preoperatively. The deterioration in HRQoL sustained also twelve months after the fracture. The use of the descriptive profile of the EQ-5D is useful when assessing quality of life after hip fracture surgery.

## Abbreviations

VAS, visual analalogue scale; HRQoL, health related quality of life; EQ-5D, EuroQol – 5 dimensions questionnaire; PROM, patient reported outcome measures; NHFR, Norwegian Hip Fracture Register; FFN, fracture of femoral neck; ASA, American Society of Anaesthesiologists.

## Additional files

Additional file 1: Descriptive profile of the 5 dimensions of the EQ-5D – different hip fractures. Description of data: Preoperative and postoperative distribution of the descriptive profile of the EQ-5D according to fracture type and length of follow-up. All patients included. (DOCX 18 kb) Additional file 2: Descriptive profile of the 5 dimensions of the EQ-5D – different age groups. Description of data: Preoperative and postoperative distribution of the descriptive profile of the EQ-5D according to age group and length of follow-up. All patients included. (DOCX 21 kb)
