# Supplementary material for: Quality of life following hip fractures: results from the Norwegian hip fracture register
Source: BMC Musculoskelet Disord. 2016 Jul 7;17:265. doi: 10.1186/s12891-016-1111-y (PMC4936302; doi:10.1186/s12891-016-1111-y)
Supplement: Additional file 1: — Descriptive profile of the 5 dimensions of the EQ-5D – different hip fractures. Description of data: Preoperative and postoperative distribution of the descriptive profile of the EQ-5D according to fracture type and length of follow-up. All patients included. (DOCX 18 kb) [file 12891_2016_1111_MOESM1_ESM.docx]

**Table 4 Descriptive profile of the 5 dimensions of EQ-5D – different hip fractures.** Preoperative and postoperative distribution of the descriptive profile of the EQ-5D according to fracture type and length of follow-up. All patients included.

|  | **__Preoperatively__** | | |  | **__4 months postop._** | | |  | **_12 months postop._** | | |  |
| --- | --- | --- | --- | --- | --- | --- | --- | --- | --- | --- | --- | --- |
|  | **FFN*** | **Troch** | **Subtr** | **p-value**^†^ | **FFN*** | **Troch** | **Subtr** | **p-value**^†^ | **FFN*** | **Troch** | **Subtr** | **p-value**^†^ |
| Mobility |  |  |  | <0.001 |  |  |  | <0.001 |  |  |  | <0.001 |
| Level 1 (%) | 65.3 | 58.6 | 62.7 |  | 25.1 | 13.4 | 12.3 |  | 37.0 | 23.6 | 24.8 |  |
| Level 2 (%) | 33.8 | 40.0 | 35.7 |  | 72.4 | 83.2 | 85.0 |  | 59.9 | 73.1 | 71.9 |  |
| Level 3 (%) | 0.8 | 1.4 | 1.6 |  | 2.5 | 3.5 | 2.6 |  | 3.1 | 3.3 | 3.3 |  |
| Self-care |  |  |  | <0.001 |  |  |  | <0.001 |  |  |  | <0.001 |
| Level 1 (%) | 80.0 | 74.0 | 77.0 |  | 56.8 | 47.2 | 49.0 |  | 63.4 | 53.7 | 56.8 |  |
| Level 2 (%) | 16.0 | 21.0 | 18.9 |  | 34.4 | 41.5 | 40.9 |  | 28.0 | 35.9 | 33.6 |  |
| Level 3 (%) | 3.9 | 5.0 | 4.1 |  | 8.8 | 11.3 | 10.1 |  | 8.6 | 10.3 | 9.6 |  |
| Usual activities |  |  |  | <0.001 |  |  |  | <0.001 |  |  |  | <0.001 |
| Level 1 (%) | 63.3 | 55.6 | 60.7 |  | 29.8 | 20.1 | 20.3 |  | 37.8 | 27.0 | 29.0 |  |
| Level 2 (%) | 27.9 | 32.9 | 30.4 |  | 52.3 | 56.6 | 56.9 |  | 44.9 | 49.6 | 52.0 |  |
| Level 3 (%) | 8.8 | 11.5 | 8.9 |  | 17.9 | 23.3 | 22.7 |  | 17.3 | 23.3 | 23.3 |  |
| Pain / discomfort |  |  |  | <0.001 |  |  |  | <0.001 |  |  |  | <0.001 |
| Level 1 (%) | 64.8 | 59.5 | 61.4 |  | 29.6 | 20.6 | 18.6 |  | 38.8 | 28.9 | 29.1 |  |
| Level 2 (%) | 30.9 | 34.7 | 31.9 |  | 62.7 | 68.6 | 71.9 |  | 55.1 | 63.2 | 62.1 |  |
| Level 3 (%) | 4.3 | 5.8 | 6.7 |  | 7.7 | 10.8 | 9.5 |  | 6.1 | 7.9 | 8.8 |  |
| Anxiety / depression |  |  |  | 0,378 |  |  |  | 0.001 |  |  |  | <0.001 |
| Level 1 (%) | 74.9 | 72.8 | 72.9 |  | 64.5 | 60.7 | 55.2 |  | 65.8 | 60.4 | 61.4 |  |
| Level 2 (%) | 22.6 | 24.3 | 24.6 |  | 31.7 | 34.4 | 41.0 |  | 30.9 | 35.7 | 35.5 |  |
| Level 3 (%) | 2.6 | 2.9 | 2.5 |  | 3.8 | 4.9 | 3.8 |  | 3.3 | 4.0 | 3.1 |  |

*FNF, femoral neck fracture

^†^ Pearson chi-squared test
