# Supplementary material for: Quality of life following hip fractures: results from the Norwegian hip fracture register
Source: BMC Musculoskelet Disord. 2016 Jul 7;17:265. doi: 10.1186/s12891-016-1111-y (PMC4936302; doi:10.1186/s12891-016-1111-y)
Supplement: Additional file 2: — Descriptive profile of the 5 dimensions of the EQ-5D – different age groups. Description of data: Preoperative and postoperative distribution of the descriptive profile of the EQ-5D according to age group and length of follow-up. All patients included. (DOCX 21 kb) [file 12891_2016_1111_MOESM2_ESM.docx]

**Table 5 Descriptive profile of the 5 dimensions of EQ-5D – different age groups**. Preoperative and postoperative distribution of the descriptive profile of the EQ-5D according to age groups and length of follow-up. All patients included.

|  | **_Preoperatively__** | | | | **4 months postop.__** | | | | **_12 months postop.__** | | | |
| --- | --- | --- | --- | --- | --- | --- | --- | --- | --- | --- | --- | --- |
|  | **<70** | **70-79** | **80+** | **p-value*** | **<70** | **70-79** | **80+** | **p-value*** | **<70** | **70-79** | **80+** | **p-value*** |
| Mobility |  |  |  | <0.001 |  |  |  | <0.001 |  |  |  | <0.001 |
| Level 1 (%) | 75.5 | 66.5 | 55.1 |  | 29.6 | 23.0 | 13.9 |  | 43.8 | 34.7 | 23.7 |  |
| Level 2 (%) | 23.3 | 32.6 | 43.8 |  | 69.2 | 75.1 | 82.1 |  | 55.1 | 63.4 | 71.6 |  |
| Level 3 (%) | 1.2 | 0.9 | 1.2 |  | 1.2 | 7.9 | 4.1 |  | 1.1 | 1.9 | 4.7 |  |
| Self-care |  |  |  | <0.001 |  |  |  | <0.001 |  |  |  | <0.001 |
| Level 1 (%) | 88.4 | 82.1 | 70.7 |  | 69.6 | 61.1 | 41.0 |  | 76.9 | 67.2 | 47.7 |  |
| Level 2 (%) | 9.5 | 14.8 | 23.4 |  | 27.6 | 32.8 | 44.3 |  | 20.6 | 27.3 | 38.2 |  |
| Level 3 (%) | 2.2 | 3.1 | 5.9 |  | 2.8 | 6.1 | 14.7 |  | 2.5 | 5.5 | 14.1 |  |
| Usual activities |  |  |  | <0.001 |  |  |  | <0.001 |  |  |  | <0.001 |
| Level 1 (%) | 75.3 | 66.9 | 50.3 |  | 37.1 | 31.0 | 17.4 |  | 48.6 | 39.2 | 23.4 |  |
| Level 2 (%) | 18.8 | 26.2 | 36.7 |  | 52.5 | 54.3 | 55.0 |  | 43.6 | 48.0 | 48.4 |  |
| Level 3 (%) | 5.9 | 6.9 | 12.9 |  | 10.4 | 14.7 | 27.5 |  | 7.8 | 12.8 | 28.2 |  |
| Pain / discomfort |  |  |  | <0.001 |  |  |  | <0.001 |  |  |  | <0.001 |
| Level 1 (%) | 70.5 | 63.7 | 58.3 |  | 20.8 | 25.6 | 27.0 |  | 27.2 | 34.2 | 37.3 |  |
| Level 2 (%) | 23.5 | 31.4 | 36.9 |  | 69.7 | 65.6 | 64.0 |  | 64.6 | 59.0 | 56.1 |  |
| Level 3 (%) | 6.1 | 4.9 | 4.8 |  | 9.5 | 8.8 | 9.0 |  | 8.2 | 6.8 | 6.6 |  |
| Anxiety / depression |  |  |  | <0.001 |  |  |  | <0.001 |  |  |  | <0.001 |
| Level 1 (%) | 81.3 | 76.4 | 69.6 |  | 71.0 | 65.0 | 58.0 |  | 72.1 | 65.5 | 58.7 |  |
| Level 2 (%) | 16.4 | 21.0 | 27.5 |  | 25.8 | 30.7 | 37.3 |  | 25.0 | 31.2 | 37.4 |  |
| Level 3 (%) | 2.3 | 2.6 | 2.9 |  | 3.3 | 4.3 | 4.7 |  | 2.9 | 3.3 | 3.9 |  |

* Pearson chi-squared test
